# Probing tissue transglutaminase mediated vascular smooth muscle cell aging using a novel transamidation-deficient *Tgm2*-C277S mouse model

**DOI:** 10.1038/s41420-021-00543-8

**Published:** 2021-07-29

**Authors:** Huilei Wang, James Chen, Sandeep Jandu, Sean Melucci, William Savage, Kavitha Nandakumar, Sara K. Kang, Sebastian Barreto-Ortiz, Alan Poe, Shivam Rastogi, Maria Bauer, Jochen Steppan, Lakshmi Santhanam

**Affiliations:** 1 Department of Biomedical Engineering, Johns Hopkins University, Baltimore, MD USA; 2 Department of Anesthesiology & Critical Care Medicine, Johns Hopkins University, Baltimore, MD USA; 3 Department of Chemical and Biomolecular Engineering, Johns Hopkins University, Baltimore, MD USA

**Keywords:** Vascular diseases, Ageing

## Abstract

Tissue transglutaminase (TG2), a multifunctional protein of the transglutaminase family, has putative transamidation-independent functions in aging-associated vascular stiffening and dysfunction. Developing preclinical models will be critical to fully understand the physiologic relevance of TG2’s transamidation-independent activity and to identify the specific function of TG2 for therapeutic targeting. Therefore, in this study, we harnessed CRISPR-Cas9 gene editing technology to introduce a mutation at cysteine 277 in the active site of the mouse *Tgm2* gene. Heterozygous and homozygous *Tgm2*-C277S mice were phenotypically normal and were born at the expected Mendelian frequency. TG2 protein was ubiquitously expressed in the *Tgm2*-C277S mice at levels similar to those of wild-type (WT) mice. In the *Tgm2*-C277S mice, TG2 transglutaminase function was successfully obliterated, but the transamidation-independent functions ascribed to GTP, fibronectin, and integrin binding were preserved. In vitro, a remodeling stimulus led to the significant loss of vascular compliance in WT mice, but not in the *Tgm2*-C277S or TG2^−/−^ mice. Vascular stiffness increased with age in WT mice, as measured by pulse-wave velocity and tensile testing. *Tgm2*-C277S mice were protected from age-associated vascular stiffening, and TG2 knockout yielded further protection. Together, these studies show that TG2 contributes significantly to overall vascular modulus and vasoreactivity independent of its transamidation function, but that transamidation activity is a significant cause of vascular matrix stiffening during aging. Finally, the *Tgm2*-C277S mice can be used for in vivo studies to explore the transamidation-independent roles of TG2 in physiology and pathophysiology.

## Introduction

Age-associated vascular stiffening is a multifactorial process that involves alterations to both vascular extracellular matrix (ECM) and vascular smooth muscle cells (VSMCs)—the primary load-bearing elements in the aorta [1]. Prior studies revealed a central role for the enzyme tissue transglutaminase (TG2) in vascular stiffening, and established that TG2 contributes to vascular stiffening both by promoting ECM deposition and by regulating VSMC tone/stiffness [2–4].

TG2, the most widely studied member of the transglutaminase superfamily, is a multifunctional protein with a complex biochemical profile [5–7]. The pathological role of its classical transglutaminase function, wherein it catalyzes the Ca^2+^-dependent crosslinking of primary amine groups to glutamine residues, is highly studied. In the ECM, TG2 crosslinks matrix proteins to form stable isopeptide bonds between glutamine and lysine residues of substrate proteins [5–7]. The resulting covalent bonds are resistant to proteolytic cleavage, and serve to stabilize and provide mechanical strength to the ECM. The transamidation-dependent ECM remodeling/deposition function of TG2 has a well-established role in fibrosis [8–10], cancers [11, 12], and neurodegenerative diseases [13–16]. The central role of TG2’s transamidation function in this diverse set of disease processes has resulted in a keen interest to develop selective and specific inhibitors that can be deployed in vivo to interrupt its transamidation reaction [17–19]. In the vasculature, TG2 inhibition or depletion protects against age-associated vascular stiffening [2, 4, 20] and delays resistance vessel remodeling induced by hypertensive and vasoconstrictive stimuli [21–23]. Thus, TG2’s transamidation function has an established role in vascular remodeling and stiffening. However, further studies have revealed that TG2 also regulates vascular stiffness independent of its crosslinking function [3]. This property could be ascribed to either its G-protein function, wherein as Gh-alpha, TG2 signals via α-adrenergic receptors and PLCδ to promote vascular contractility/vasomotor tone [24–27], or to its adhesive function, wherein TG2 facilitates and stabilizes cell–ECM contacts by interacting with syndecans 2/4 [28–30], fibronectin, and integrins [31–33] at the cell surface. In addition to its effects on vascular stiffness and function, TG2 has recently been shown to have transamidation-independent functions that contribute to pathogenesis and progression of other diseases. In ovarian cancer, for example, inhibition of the TG2–fibronectin interaction was shown to reduce metastasis [34–36]. In addition, the GTP-binding domain, but not the catalytic domain, is postulated to be essential for epithelial-to-mesenchymal transition of mammary epithelial cells [37]. These provocative findings underscore the importance of investigating the full (patho)-physiological scope of the transamidation-independent functions of TG2 in preclinical models. Thus, in this study, we generated a novel mouse model in which we targeted the mouse *Tgm2* gene that encodes the TG2 protein by CRISPR-Cas9 gene editing technology to generate a point mutation at cysteine 277 of the active site. The resulting *Tgm2*-C277S﻿ mice express a TG2 protein that is transamidation-deficient but retains its other functions. These mice were used to isolate the crosslinking-independent functions of TG2 in vivo and investigate their relevance to vascular aging.

## Results

### Successful introduction of a point mutation at the active site C277 in mouse *Tgm2* gene

**Fig. 1:**
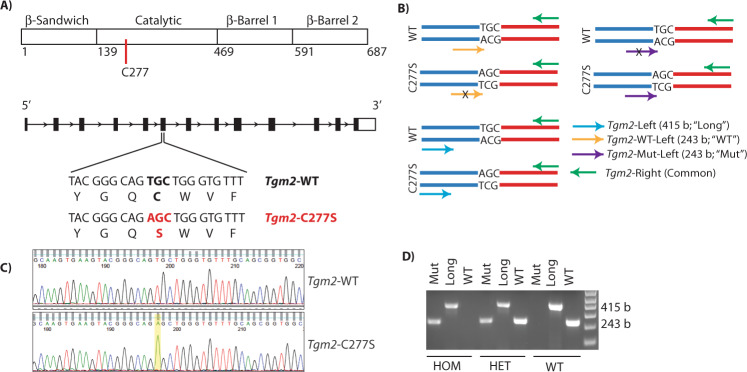
CRISPR/Cas9 modification of active site cysteine in the mouse *Tgm2* gene. **A** Schematic of TG2 protein and *Mus musculus* *Tgm2* gene. **B** Schematic illustrating the amplification refractory mutation system (ARMS) PCR assay for genotyping the *Tgm2*-C277S mouse model. **C** Sanger sequence alignment of PCR amplicon from the founder mouse with that of the WT mouse *Tgm2* gene. The point mutation corresponding to the C-to-S mutation is highlighted. **D** Representative ethidium bromide-stained agarose gel showing DNA genotyping results for a homozygous C277S mutant mouse (HOM), heterozygous mutant mouse (HET), and WT mouse.

**Fig. 2:**
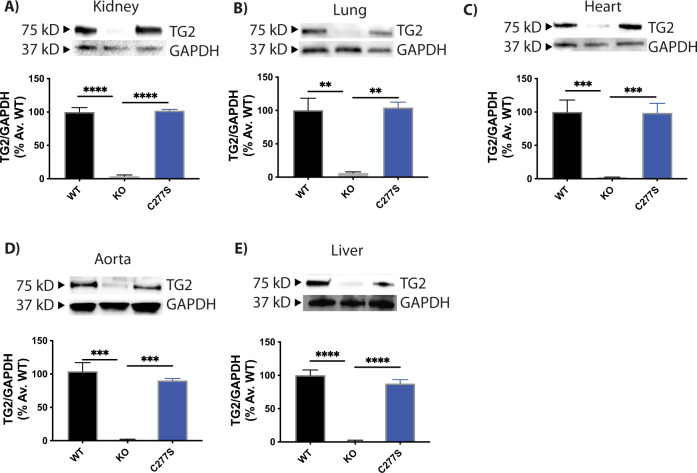
*Tgm2*-C277S mice express robust levels of TG2. Representative western blots and corresponding densitometry data showing TG2 expression in young WT and *Tgm2*-C277S littermate mice. **A** Kidney. **B** Lung. **C** Heart. **D** Aorta. **E** Liver. Age-matched TG2^−/−^ (KO) mice were used as controls. *n* = 8 per group.

The mouse *Tgm2* gene (NC_000068.7) is on chromosome 2, and contains 13 exons and 12 introns (Fig. 1A). PCR analysis of tail DNA followed by Sanger sequencing of the purified amplicon flanking the mutation site revealed successful introduction of the T-to-A point mutation encoding the active site cysteine, and was used to identify the founders (Fig. 1B). The mutation was further confirmed with an amplification refractory mutation system (ARMS) PCR assay of the tail DNA (Fig. 1C, D). We successfully identified a founder female mouse bearing the C277S point mutation and generated a colony of *Tgm2*-C277S ﻿mice. The mutant *Tgm2*-C277S mice were viable, born at the expected Mendelian frequency when heterozygous mutant mice were bred, and were of normal size and weight. The homozygous mice bred normally and did not display any complications with parturition. TG2 expression in the liver, kidney, lung, heart, and aorta was similar to that in wild-type (WT) littermates (Fig. 2). Though we noticed a trend toward decreased TG2 expression in the aorta and liver of *Tgm2*-C277S mice, the data did not reach statistical significance. Samples from TG2^−/−^ mice were used as negative controls for TG2 expression, and GAPDH was used as a loading control. Germline deletion of TG2 was reported to result in compensatory expression of TG1 and TG4 in the aorta [38, 39], and thus we tested if this also occurs in the *Tgm2*-C277S mice. No compensatory expression of TG1 or TG4 was noted in the *Tgm2*-C277S mice and WT littermates (data not shown).

### *Tgm2*-C277S protein lacks transamidation activity but retains GTP, fibronectin, and integrin β1 binding

**Fig. 3:**
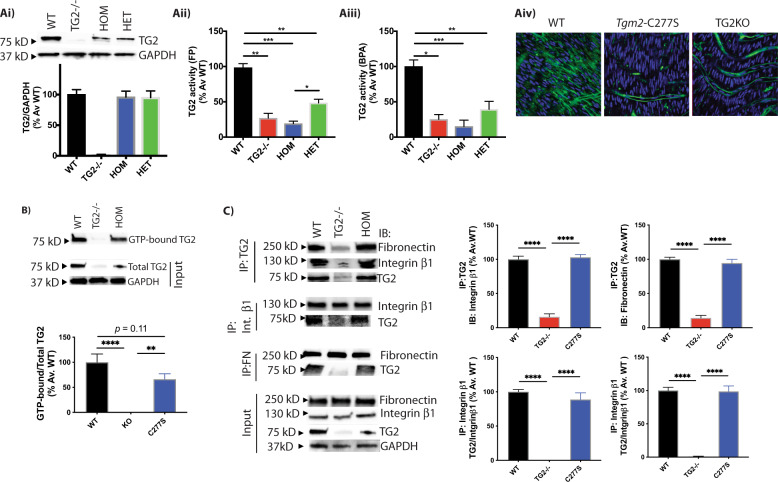
TG2 crosslinking function is lost but GTP, integrin, and fibronectin interactions are preserved in *Tgm2*-C277S mice. **A** (i) Representative western blot and densitometry analysis showing that TG2 expression levels in aortic homogenates of *Tgm2*-C277S heterozygous (HET) and homozygous (HOM) mice are similar to those in littermate WT mice. Age-matched TG2^−/−^ mice were used as controls (*n* = 8 per group). TG2 activity was significantly lower in *Tgm2*-C277S mice, as measured by (ii) crosslinking of FITC-cadaverine and *N*,*N*′-dimethylcasein determined using fluorescence polarization (FP) and (iii) biotin(amido)pentylamine (BPA) incorporation in intact aortic rings by dot blotting in the presence of DTT (*n* = 8 per group; *p < 0.05, **p < 0.01, ***p < 0.001 by one-way ANOVA with Bonferroni post hoc analysis). (iv) Representative en face confocal microscopy images show TG2-dependent incorporation of FITC-cadaverine into the aortic media of *Tgm2*-C277S mice and WT littermates in the presence of DTT, a TG2 activator. Age-matched TG2^−/−^ (KO) mice were used as controls. Blue = DAPI; green = FITC-cadaverine; *n* = 5 per group. **B** Representative western blots of WT and *Tgm2*-C277S mouse liver show GTP binding of TG2; TG2^−/−^ mice were used as controls. **C** Representative western blots of WT and *Tgm2*-C277S mouse liver show co-immunoprecipitation of TG2 with integrin β1 and fibronectin and vice versa. Blots showing total expression of these proteins are shown for reference. (*n* = 5 mice per group; **p < 0.01; ****p < 0.0001 by ordinary one-way ANOVA with Bonferroni post hoc analysis).

We first used biochemical assays to evaluate the various functions of TG2 from WT and *Tgm2*-C277S mice; tissue specimens from TG2^−/−^ mice served as negative controls. The transamidation function of TG2 was measured in mouse aorta by three approaches as described in the “Methods” section. Heterozygous specimens exhibited a 75% loss of TG2 activity when compared to specimens from WT littermates. Although TG2 protein abundance was similar to WT littermates, TG2 transamidation activity in the aortic homogenates of homozygous *Tgm2*-C277S specimens was ~85% less than that in WT samples and statistically similar to that observed in TG2^−/−^ mice (Fig. 3Ai, ii). Ex vivo activity assays in intact aorta revealed similar loss of TG2 transamidation function in *Tgm2*-C277S specimens despite the addition of dithiothreitol (DTT) to elicit maximal TG2 transamidation activity (Fig. 3Aiii, iv). GTP binding by TG2 was similar in *Tgm2*-C277S and WT genotypes, with a trend towards a modest decrease in binding by *Tgm2-*C277S protein (Fig. 3B). TG2 interactions with fibronectin and integrin β1 (CD29) were similar in WT and *Tgm2*-C277S homozygous mice, as evaluated by co-immunoprecipitation from liver homogenates (Fig. 3C).

### Cell and tissue responses ascribed to TG2’s crosslinking function are impaired in *Tgm2*-C277S mice

**Fig. 4:**
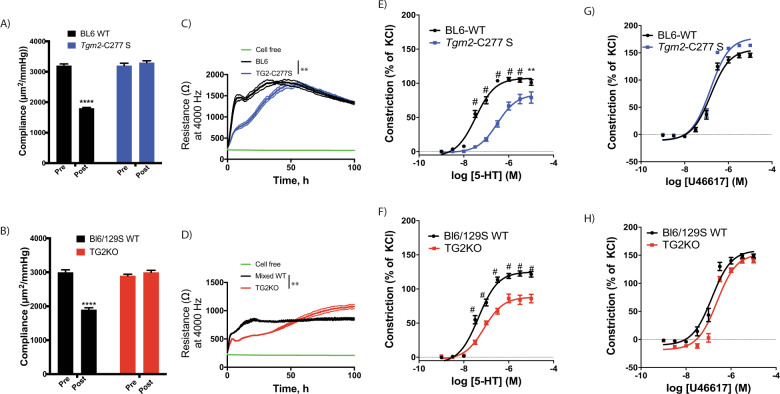
Loss of TG2 crosslinking activity alters functional responses of cells and tissue. **A**, **B** Compliance of carotid artery from *Tgm2*-C277S and WT littermate mice (**A**), and from age-matched TG2^−/−^ (KO) and WT Bl6/129S mice (**B**) before and after a remodeling stimulus (*n* = 8 per cohort; ****p < 0.0001 by Student’s *t* test). **C**, **D** Vascular smooth muscle cell (VSMC) proliferation on collagen I-coated plasticware (*n* = 5 per group; **p < 0.01 by one-way repeated measures ANOVA). **E**, **F** Contraction response of aortic rings from young *Tgm2*-C277S and WT littermate mice (**E**), and from age-matched TG2^−/−^ (KO) and WT Bl6/129S mice (**F**) in response to increasing concentrations of serotonin (5-HT; *n* = 12 per group; ^#^*p* < 0.01; **p < 0.01 vs. corresponding WT at same agonist concentration by one-way ANOVA with Bonferroni post hoc analysis). **G**, **H** Contraction response of aortic rings from young *Tgm2*-C277S and WT littermate mice (**G**), and age-matched TG2^−/−^ (KO) and WT Bl6/129S mice (**H**) in response to increasing concentrations of U46619 (*n* = 12 per group).

We next examined whether loss of TG2’s crosslinking function results in the loss of functional responses ascribed to transamidation in cells, and tissue specimens derived from *Tgm2*-C277S mice. We used age-matched TG2^−/−^ mice on a Bl6/129S mixed background served as negative controls. These mice also provided a benchmark for complete depletion of all TG2 functions. Where relevant, age-matched Bl6/129S mice served as WT controls for the TG2^−/−^ mice because the vasoreactivity and vascular mechanics of these mice differ markedly from those of C57Bl/6J mice, the background strain for the *Tgm2*-C277S mice [40]. The early phase of vascular remodeling in response to vasoconstrictive or hypertensive stimuli is dependent on the TG2 transamidation function [22, 41]. Therefore, we first examined the remodeling of carotid artery segments in response to an ex vivo remodeling stimulus composed of vasoconstriction with phenylephrine and transluminal pressure elevation to 100 mmHg. Before the stimulus was applied, the passive (Ca^2+^-free) compliance of carotid arteries from young (3–4-month old) WT, *Tgm2*-C277S, and TG2^−/−^ mice was similar. After an 8-h remodeling period, compliance was significantly decreased in segments from WT mice, but not in those from *Tgm2*-C277S mice or TG2^−/−^ mice (Fig. 4A, B).

TG2 is known to crosslink collagen I and facilitate the proliferation of various cell types, including VSMCs [42, 43]. Therefore, we next compared the proliferation of aortic VSMCs isolated from young (3–4-month old) *Tgm2*-C277S homozygous mutant mice to those from WT littermates (Fig. S1) on collagen I-coated surfaces. VSMCs from age-matched TG2^−/−^ mice on a Bl6/129S background served as controls, and were compared with age-matched Bl6/129S WT mice (Fig. S1). Proliferation was significantly delayed in VSMCs from both *Tgm2*-C277S and TG2^−/−^ mice, when compared with that in cells from the corresponding WT mice (Fig. 4C, D).

Finally, we examined serotonin (5-HT)-induced vasoconstriction, which is postulated to occur as a result of intracellular protein serotonylation by TG2-mediated transamidation [44, 45]. 5-HT-induced constriction was markedly attenuated in aortic rings from young (3–4-month old) *Tgm2*-C277S mice, when compared to that of rings from WT littermate mice (Fig. 4E). Similarly, 5-HT-induced constriction was significantly less in the aortic rings from TG2^−/−^ mice than in those from their corresponding WT mice (Fig. 4F). In contrast, vasoconstriction in response to increasing concentrations of U46619, a prostaglandin analog, which occurs independent of TG2, was similar in all cohorts (Fig. 4G, H).

### Functional effects of GTP, fibronectin/integrin binding are preserved in the aorta and VSMCs of *Tgm2*-C277S mice

**Fig. 5:**
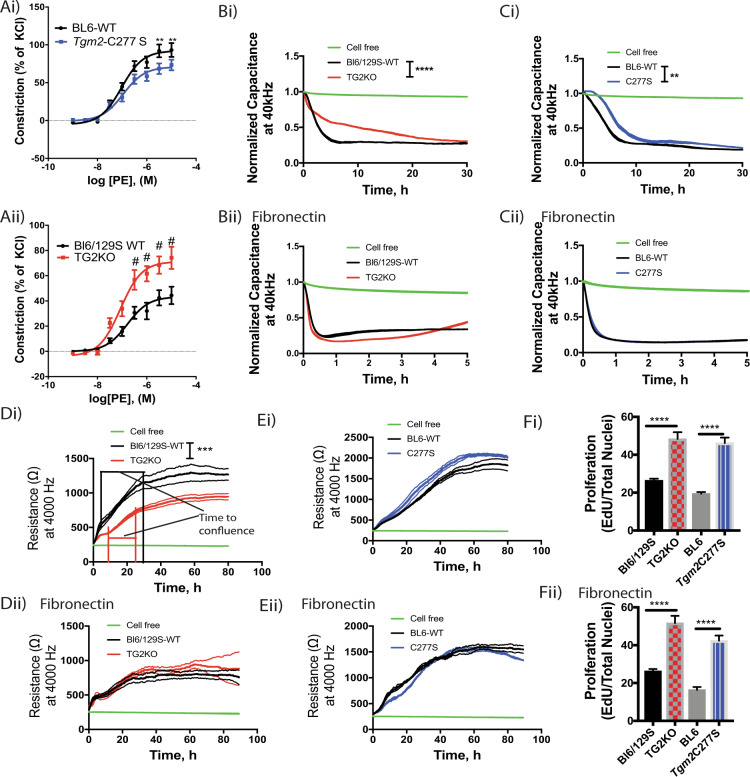
Functional effects of GTP, fibronectin/integrin binding are preserved in the aorta and vascular smooth muscle cells (VSMCs) of *Tgm2*-C277S mice. **A**, **B** Phenylephrine (PE)-induced contractility of aortic rings from *Tgm2*-C277S and WT littermate mice (**Ai**), and from age-matched TG2^−/−^ KO (**Aii**) and Bl6/129S WT mice (*n* = 8 animals per group, **p < 0.01, ^#^*p* < 0.001 by one-way ANOVA with Bonferroni post hoc analysis). **B** Adhesion and spreading of VSMCs from TG2^−/−^ (KO) and age-matched Bl6/129S WT mice on cell culture plastic (i) and fibronectin-coated cell culture plastic (ii). (*n* = 6; ****p < 0.0001, by one-way re*p*eated measures ANOVA with Bonferroni post hoc analysis). **C** Adhesion and spreading of VSMCs from *Tgm2*-C277S and WT littermate mice on cell culture plastic (i) and fibronectin-coated cell culture plastic (ii). (*n* = 6; **p < 0.01 by one-way repeated measures ANOVA with Bonferroni post hoc analysis). **D** Proliferation of VSMCs from TG2^−/−^ (KO) and age-matched Bl6/129S WT mice on cell culture plastic (i) and fibronectin-coated cell culture plastic (ii). (*n* = 6; ***p < 0.001 by one-way repeated measures ANOVA with Bonferroni post hoc analysis). **E** Proliferation of VSMCs from *Tgm2*-C277S and littermate WT mice on cell culture plastic (i) and fibronectin-coated cell culture plastic (ii). (*n* = 6; **p < 0.01 by one-way repeated measures ANOVA with Bonferroni *p*ost hoc analysis). **F** DNA synthesis proliferation assay on cell culture plastic (i) and fibronectin-coated surface (ii) (*n* = 6; ****p < 0.0001 by Student’s *t* test).

Next, we examined whether cell and tissue functions attributed to GTPase activity and fibronectin/integrin binding are preserved in the *Tgm2*-C277S mice. The GTPase function of TG2 promotes α1(B)-adrenoreceptor-dependent vasoconstriction in a bimodal fashion [46]. Therefore, we evaluated the contractility of aortae from young (3–4-month old) *Tgm2-*C277S and WT littermate mice in response to increasing concentrations of the α1(B)-adrenoreceptor agonist phenylephrine. Again, age-matched TG2^−/−^ mice and Bl6/129S WT mice were used as controls. Phenylephrine-induced vasoconstriction was only modestly lower in *Tgm2*-C277S rings than in those from WT littermates (Fig﻿. 5Ai), but it was significantly augmented in rings from TG2^−/−^ mice, when compared with those from age-matched WT mice (Fig. 5Aii).

Cell adhesion/spreading is facilitated by cell surface TG2 independent of its crosslinking function and should be preserved in cells from *Tgm2*-C277S mice. Therefore, we next compared the adhesion and spreading of aortic VSMCs isolated from young *Tgm2*-C277S mice to that of VSMCs isolated from WT littermates (Fig. S1). VSMCs from age-matched TG2^−/−^ and Bl6/129S WT mice were used as controls (Fig. S1). VSMC adhesion on cell culture plasticware was significantly delayed in cells lacking TG2 protein (Fig. 5Bi), but was rescued with the provision of exogenous fibronectin (Fig. 5Bii). *Tgm2*-C277S mutation delayed cell adhesion to a modest degree, but again, provision of fibronectin fully restored cell adhesion and spreading dynamics to that of WT cells (Fig. 5Ci, ii). The fibronectin used to coat the cell culture surface was free of TG2, as determined by western blotting (Fig. S2). Cell surface TG2 is also shown to regulate cell proliferation, independent of its crosslinking function. In the TG2^−/−^ VSMCs, we found that while the onset of proliferation (reflected by a rapid increase in transwell resistance) was strikingly delayed compared to that of WT counterparts, the cells reached confluence (stable plateau) earlier, indicating a faster proliferation rate. Again, exogenous fibronectin fully restored TG2^−/−^ VSMC proliferation to levels comparable to those of WT cells (Fig. 5Di, ii). In contrast, proliferation of *Tgm2*-C277S VSMCs was modestly higher than that of WT VSMCs on cell culture plastic, but similar on fibronectin-coated surfaces (Fig. 5Ei, ii). Interestingly, DNA synthesis, as examined by EdU incorporation, was higher in both TG2^−/−^ and *Tgm2*-C277S, when compared with corresponding WT VSMCs, independent of fibronectin (Fig. 5Fi, ii), revealing an overall higher proliferation rate in the absence of TG2’s crosslinking function.

### TG2 mediates vascular stiffening in aging by a dual mechanism

**Fig. 6:**
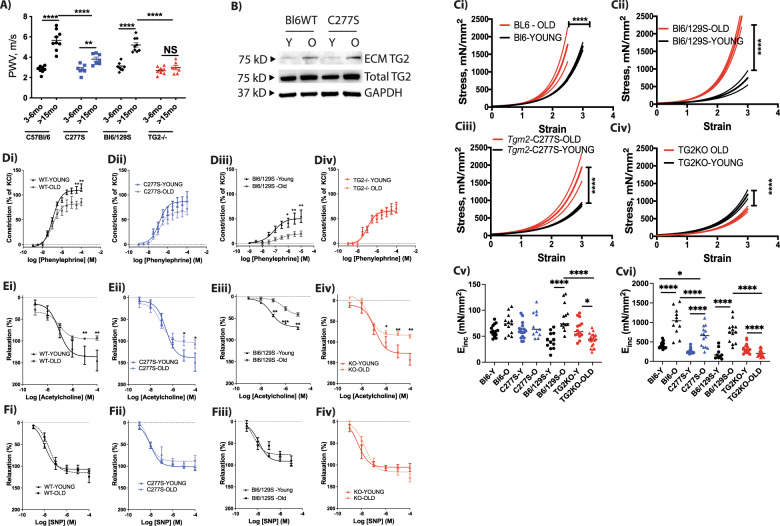
TG2 mediates vascular stiffening in aging by a dual mechanism. **A** Pulse-wave velocity (PWV) in young and old *Tgm2*-C277S and WT littermate mice; age-matched TG2^−/−^ (KO) mice are shown for reference. (*n* = 10 per group; **p < 0.01, ****p < 0.0001 by one-way ANOVA with Bonferroni post hoc correction). **B** Representative western blot of decellularized aortae from young and old *Tgm2*-C277S and WT littermate mice. Total TG2 and GAPDH expression are shown as references (*n* = 5 per cohort). **C** Tensile testing of aortae from young and old WT (i), *Tgm2*-C277S (iii), Bl6/129S WT (ii), and TG2^−/−^ (KO) (iv) mice. Data are shown as mean (solid line) ± standard deviation (dotted lines). (*n* = 8–12 mice per group; ****p < 0.0001 by two-way ANOVA with Bonferroni post hoc analysis). Bar graphs show Incremental elastic modulus (*E*_inc_) at a strain of 0.5 (v), and at a strain of 1.8 (vi). (*n* = 8–12 mice, two samples per mouse; *p < 0.05, ****p < 0.0001 by ordinary one-way ANOVA with Bonferroni post hoc analysis). **D** Contraction response of aortic segments in response to increasing concentrations of phenylephrine in WT (i), *Tgm2*-C277S (ii), Bl6/129S (iii), and TG2^−/−^ (iv) (*n* = 8 mice per group; **p < 0.01 vs. young at same concentration by two-way ANOVA). **E** Endothelium-dependent relaxation of phenylephrine-preconstricted rings in response to increasing concentrations of acetylcholine in WT (i), *Tgm2*-C277S (ii), Bl6/129S (iii), and TG2^−/−^ (iv) (*n* = 8 mice per group; **p < 0.01 vs. young at same concentration by two-way ANOVA). **F** Endothelium-independent relaxation of phenylephrine-preconstricted aortic rings in response to increasing concentrations of sodium nitroprusside (SNP) in WT (i), *Tgm2*-C277S (ii), Bl6/129S (iii), and TG2^−/−^ (iv).

Subtle changes to both vascular ECM and VSMC tone/stiffness propagate as measurable changes in in vivo vascular stiffness. TG2 is associated with VSMC contractility and stiffness, as well as ECM remodeling and stiffening, and can thus contribute to vascular stiffening through these two distinct mechanisms [3, 47]. Prior studies have shown that pulse-wave velocity (PWV), an index of vascular stiffness, is significantly elevated and plateaus by 15 months of age in mice and precedes the onset of systolic hypertension in rodent models [4, 48]. Therefore, we investigated the role of TG2 in age-associated vascular stiffening using young (3–6-month old) and old (>15-month old) mice. PWV was significantly higher in the old WT mice than in the young WT mice, for both C57Bl/6 and Bl6/129S strains. In the *Tgm2*-C277S mice, PWV increased with age, but to a lesser extent than that observed in littermate WT mice. On the other hand, TG2^−/−^ mice were completely protected from age-associated increases in PWV (Fig. 6A), when compared with corresponding Bl6/129S WT mice. TG2 expression was augmented with age in the decellularized aortic matrix from both the C57Bl/6 WT and *Tgm2*-C277S mice (Fig. 6B). We next determined the mechanical properties of the aorta by tensile testing. Aging was associated with significant aortic stiffening in both the C57Bl/6 and Bl6/129S WT mice (Fig. 6Ci, ii). Stiffening also occurred with aging in *Tgm2*-C277S mouse aortae, but the magnitude of increase was less than that in WT mice (Fig. 6Ciii). Surprisingly, aortae from old TG2^−/−^ mice were less stiff than those from young TG2^−/−^ mice (Fig. 6Civ). We also compared incremental elastic modulus (*E*_inc_) at a strain of 0.5, representing elastin deformation, and at a strain of 1.8, representing collagen deformation. At low strain, *E*_inc_ was unchanged with age in the C57Bl/6 WT and littermate *Tgm2*-C277S mice, increased in the Bl6/129S mice with age, and decreased in the TG2KO mice with age (Fig. 6Cv). A significant age-related increase in *E*_inc_ was noted at the higher strain in the C57Bl/6 and Bl6/129S WT mice (Fig. 6Cvi). *Tgm2*-C277S mice also exhibited a significant increase in stiffness with age, but to a lesser extent than the Bl6 WT littermates. In contrast, at the higher strain, *E*_inc_ decreased with age in the TG2^−/−^ mice (Fig. 6Cvi).

**Fig. 7:**
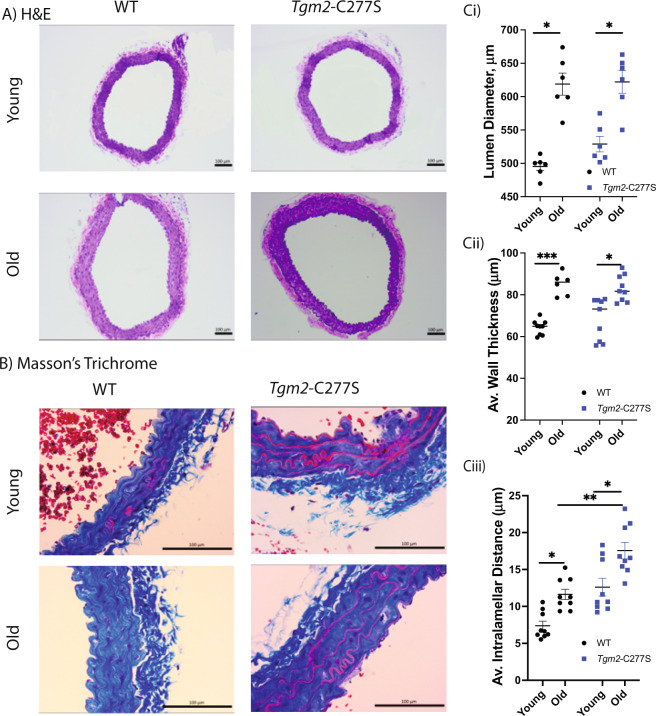
Histochemical analysis of aorta from young and old mice. **A**, **B** Representative hematoxylin and eosin (**A**) and Masson’s trichrome (**B**) staining of cross-sections of the aorta. **C** Bar graphs of Lumen diameter (i), wall thickness (ii), and intralamellar distance (iii) in aorta from old and young littermate WT and *Tgm2*-C277S mice; (*n* = 5 mice per cohort; *p < 0.05, **p < 0.01, ***p < 0.001 by ordinary one-way ANOVA with Bonferroni post hoc analysis).

We next compared changes in vascular contraction and relaxation responses by wire myography. Aging significantly impaired phenylephrine-induced vasoconstriction (Fig. 6D) in both C57Bl/6 and Bl6/129S WT mice. The aging-related change in phenylephrine-induced vasoconstriction was attenuated in the *Tgm2*-C277S mice and absent in TG2^−/−^ mice (Fig. 6D). Acetylcholine-induced endothelial-dependent vasorelaxation was significantly impaired with age in all the groups (Fig. 6E), whereas endothelial-independent vasorelaxation induced by sodium nitroprusside was unimpaired in all the groups (Fig. 6F). Histochemical analysis of aortic segments showed a marked increase in lumen diameter, wall thickness, and intralamellar distance in both old WT and *Tgm2*-C277S mice, when compared with young counterparts (Fig. 7A–C).

## Discussion

In our study, we used CRISPR-Cas9 gene editing to introduce a mutation to the mouse *Tgm2* gene that would disrupt the crosslinking function of the TG2 protein. These *Tgm2*-C277S mice expressed robust levels of TG2 protein that were modestly, but not significantly, attenuated from those of WT littermates. Biochemical activity assays revealed that the *Tgm2*-C277S mutant protein was indeed deficient in crosslinking function (Fig. 2). The loss of TG2-mediated crosslinking activity translated to a decrease in ex vivo vascular remodeling and delayed VSMC proliferation on collagen I-coated surfaces. Importantly, *Tgm2-*C277S protein retained its crosslinking-independent functions, as evidenced by the preservation of GTP, fibronectin, and integrin β1 binding and the phenylephrine-induced aortic contractility seen in mutant mice. Thus, these mice can be used to tease apart and explore the transamidation-dependent and -independent functionality of TG2.

TG2 was first identified as the protein Gh-α that mediates signaling via α-adrenoreceptors, a functionality that is considered to be transamidation independent. Therefore, we examined GTP binding by the *Tgm2*-C277S protein and found it to be modestly lower than that of native TG2, consistent with prior reports [37, 49]. As no GTPase activity assays are available for TG2, we examined phenylephrine-induced contractility as a surrogate measure of GTPase function of TG2. Aortic rings from *Tgm2-*C277S mice showed only a modest attenuation of phenylephrine-induced vasoconstriction, as is expected given the modest loss of GTP binding. In contrast, TG2 knockout significantly augmented phenylephrine-induced constriction, as shown previously [3]. These findings are consistent with the previously reported bimodal effect of TG2 on α-adrenoreceptor signaling, and confirm the functional presence of Gh-α activity in the *Tgm2*-C277S mice [46].

The second well-established crosslinking-independent function of TG2 is cell adhesion and proliferation mediated by cell surface TG2. TG2 facilitates cell adhesion by binding to integrins, fibronectin, and syndecan 2/4 [6, 29–32, 50]. Biochemical assays confirmed the direct interaction of *Tgm2*-C277S protein with fibronectin and integrin β1. We used cell adhesion assays to further confirm the functionality of this interaction in the *Tgm2*-C277S mice. Whereas cell adhesion and spreading dynamics were significantly delayed in VSMCs isolated from TG2^−/−^ mice, when compared to that of their WT counterparts, they were only modestly delayed in the *Tgm2*-C277S VSMCs. This small delay was likely due to the slightly lower *Tgm2*-C277S protein expression observed on western blots. Provision of fibronectin fully restored VSMC adhesion and spreading dynamics in both TG2^−/−^ and *Tgm2*-C277S VSMCs. We confirmed that TG2 protein is not present in the fibronectin used in these studies as a contaminant, and thus the results are ascribed to cellular TG2. These findings suggest that TG2 rescues cell adhesion in the absence or impairment of direct fibronectin–integrin interactions independent of its crosslinking function, but is dispensable when sufficient fibronectin–integrin interaction is present. Based on prior studies that established the requirement for a TG2–syndecan interaction to rescue RGD-impaired cell adhesion, we postulate that a similar mechanism exists in VSMCs. However, the presence and role of TG2–syndecan interactions in the *Tgm2*-C277S mice remains to be directly validated. Thus, our studies confirm that TG2-mediated cell adhesion is functionally present in the *Tgm2*-C277S mice.

The role of TG2 in VSMC proliferation is more intricate. In our study, we examined cell proliferation using two approaches—DNA synthesis by EdU incorporation and cell count/spreading using electrical cell-substrate impedance sensing (ECIS). VSMCs from *Tgm2*-C277S mice exhibited a higher proliferation rate, when compared to their WT counterparts by both methods and similar transwell resistance levels at plateau in the ECIS assay. VSMCs from TG2^−/−^ mice also displayed accelerated proliferation, but established a lower transwell resistance at plateau on uncoated plasticware. Interestingly, provision of fibronectin restored transwell resistance in TG2^−/−^ VSMCs, but did not restore DNA synthesis toward that of WT in TG2^−/−^ or *Tgm2*-C277S VSMCs. Our study further revealed that TG2’s transamidation function suppresses DNA synthesis/proliferation of VSMCs, but that its adhesive function promotes cell spreading and cell–cell contacts during cell proliferation, particularly in the absence of direct fibronectin–integrin interactions. In addition, TG2 crosslinking function emerged as an essential component of VSMC proliferation on collagen I. Therefore, in sum, our results indicate that TG2’s role in cell proliferation involves the interplay between its various functions and the type of substratum used in the investigation. Therefore, we conclude that in vivo, the role of TG2 in VSMC proliferation would be highly complex, given the rich compositional diversity of the in vivo ECM.

Prior studies have shown that the nonspecific transglutaminase inhibitor cystamine attenuates 5-HT-induced vasoconstriction at low concentrations, and completely blocks the response at higher concentrations (1 mg/mL) [44, 45]. Thus, 5-HT-induced vasoconstriction is postulated to occur through protein serotonylation catalyzed by TG2-mediated transamidation. We found that 5-HT-induced vasoconstriction was significantly attenuated in *Tgm2*-C277S mice, when compared with that of WT littermates. The difference was similar to that observed between TG2^−/−^ mice and age-matched WT controls. Thus, our study provides the first direct evidence for the involvement of TG2’s transamidation function in 5-HT-induced vasoconstriction. However, neither ablation of TG2’s crosslinking function (*Tgm2*-C277S mice) nor deletion of the protein itself (TG2^−/−^ mice) resulted in the complete loss of 5-HT-induced vasoconstriction, as is reported at high concentrations of cystamine [44, 45]. Thus, this study suggests that TG2 is not the sole purveyor of 5-HT signaling in the vasculature, and the effect of high cystamine concentrations on 5-HT-induced contraction likely occurs through TG2-independent mechanisms, such as soluble guanylate cyclase inhibition [51, 52].

Finally, we used these newly developed *Tgm2*-C277S mice to investigate the crosslinking-dependent and crosslinking-independent roles of TG2 in aging-associated mechanical and functional deterioration of the large compliance vessels. We first assessed PWV, an index of in vivo vascular stiffness. In Bl6/129S WT mice, aging resulted in a large increase in PWV that was completely absent in the TG2^−/−^ mice. Interestingly, though we observed a statistically significant age-associated PWV increase in *Tgm2*-C277S mice, the magnitude of change was significantly less than that in littermate WT mice. Passive mechanical stiffness also increased significantly with age in WT mice and to a lesser magnitude in *Tgm2*-C277S mice, as noted by tensile testing. Notably, the TG2^−/−^ mice exhibited a marked decrease in passive stiffness with aging. Although this decrease could be due to age-associated activation of matrix degradation pathways in the absence of matrix deposition by TG2 [53–55], this explanation is not complete, as *Tgm2*-C277S mice, which also lack the transamidation function, did not show a similar decline in mechanical modulus. Therefore, when taken together, these findings reveal a significant scaffolding role for TG2 in the vascular matrix that contributes to overall load-bearing, but is independent of crosslinking function. The importance of this role is underscored by the compensation it provides with regard to load-bearing even when crosslinking mechanisms fail to engage in vascular remodeling. With regard to vascular aging, prior studies have clearly shown that VSMC aging includes VSMC stiffening, changes in VSMC–ECM contact strength and number, and loss of functional contractility [1, 56]. These functional and mechanical changes in VSMCs are both the cause and consequence of ECM remodeling, owing to the dynamic reciprocity between cells and their ECM [1, 56]. When taken in the context of these prior studies, our findings show that (1) the *Tgm2*-C277S mice offer a novel model in which to examine VSMC aging, when uncoupled from ECM remodeling and stiffening, and (2) VSMC dysfunction in aging arises independently of ECM remodeling/stiffening, and likely precedes ECM remodeling. Importantly, preventing ECM remodeling prevents the entry of the vasculature into the vicious feed-forward cycle of VSMC dysfunction and ECM stiffening, and decelerates or halts the progression of vascular stiffening.

Aged WT mice also exhibited a marked impairment in vasoconstriction responses to phenylephrine. Loss of TG2’s crosslinking function (*Tgm2*-C277S mutation) conferred partial protection, and deletion of TG2 protein (TG2^−/−^) provided further improvement in aging-associated vasoconstriction. Endothelial-dependent relaxation was markedly attenuated in all the strains of mice, showing that endothelial dysfunction emerges independent of vascular stiffening in the aging vasculature, and indeed, could be an early first step in vascular aging. These results show that, when compared to WT mice, *Tgm2*-C277S mice were significantly protected from age-associated decline in vascular mechanics and function. The overall responses of the *Tgm2*-C277S mice were closer to those of the TG2^−/−^ mice than to those of WT. Thus, the TG2 transamidation function contributes substantially to long-term aging-associated vascular stiffening by causing ECM stiffening. The crosslinking-independent functions of TG2 make a smaller, but still significant contribution to vascular stiffening. Our findings further show that the TG2-dependent ECM remodeling and stiffening secondarily cause dysregulation of VSMC tone and behavior in the aging vessel, as a result of the dynamic reciprocity between the vascular ECM and the resident VSMCs. Lastly, our studies show that TG2 can modulate acute vascular contractility independent of its crosslinking function. This activity can play a role in hypertension-induced resistance vessel remodeling and atherosclerosis, and needs to be studied further.

In addition to cardiovascular diseases, TG2 plays a role in a diverse set of other conditions, including celiac disease, cancers (e.g., ovarian, breast, cervical, pancreatic, colorectal, liver, lung, and skin), vascular remodeling and stiffening in the systemic vasculature, pulmonary arterial hypertension, renal fibrosis, pulmonary fibrosis, and cardiac fibrosis. Classically, the transamidation function of TG2 has been implicated in these disease states, and a number of specific inhibitors that target this function have been developed [17, 57, 58]. Recent studies also have illuminated transamidation-independent functions of TG2 as important contributors to pathogenesis and disease progression. For example, in ovarian cancer, inhibition of the TG2–fibronectin interaction was shown to reduce metastasis [34, 35]. The GTP-binding domain, but not the catalytic domain, has been postulated as being essential for epithelial-to-mesenchymal transition of mammary epithelial cells [37]. Overall, our study shows that the newly developed *Tgm2*-C277S mouse model can be used to gain detailed insights into the biology and pathobiology of conditions, in which the transamidation-independent functions of TG2 have been implicated. Furthermore, combining this model with newly developed TG2-specific inhibitors will yield a comprehensive understanding of TG2’s functional repertoire in vivo and ex vivo. Finally, the *Tgm2*-C277S mouse can be used to study cellular responses when uncoupled from ECM remodeling in disease models, where TG2 serves as a primary matrix deposition pathway to gain a foundational understanding of how the dynamic reciprocity between the cells and ECM drives pathophysiological/maladaptive tissue responses.

## Materials and methods

### Animals

*Tgm2*-C277S point mutant knock-in mice on a C56Bl/6 background and WT littermates were used in this study. Age-matched TG2 knockout (TG2^−/−^) mice on a Bl6/129S mixed background were used as controls lacking all TG2 functions. As there are unique differences between the vascular responses of WT C57Bl/6 and Bl6/129S mice [40], age-matched Bl6/129S mixed background mice (Jackson Labs) were used as WT controls for the TG2^−/−^ mice. The responses of *Tgm2*-C277S mice are compared with littermate WT C57Bl/6J mice, whereas the responses of TG2^−/−^ mice are compared with Bl6/129S mixed background WT mice, in order to account for the background strains of these two distinct mouse models.

All animals were maintained in the Johns Hopkins University School of Medicine animal care facility. Animals were fed and watered ad libitum, maintained on a 12‐h light/dark cycle in a pathogen-free facility, and used with appropriate Animal Care and Use Committee approvals. Breeding pairs were composed of (1) heterozygous *Tgm2*-C277S mice (to yield WT, heterozygous, and homozygous *Tgm2*-C277S mice); (2) homozygous *Tgm2*-C277S mice (to yield *Tgm2*-C277S homozygous mice for rapid experimentation); (3) and homozygous TG2^−/−^ mice for the knockout cohort of mice. Bl6/129S WT mice were purchased from the Jackson Laboratory at 8 weeks of age and maintained until the appropriate age for use.

### *Tgm2*-C277S mice

Two guide RNAs (gRNAs) targeting the mouse *Tgm2* gene within 10–20 bases of the active site cysteine were designed with the following sequences:

gRNA 1: GTGCTGGGTGTTTGCAGCGG**TGG** and

gRNA 2: AGTGAAGTACGGGCAGTGCT**GGG**.

A homology directed repair (HDR) donor single-stranded oligonucleotide with the sequence GTCCCATGGCCTGGATTGGCAGTGTGGACATTCTGCGGCGCTGGAAGGAACACGGCTGTCAGCAAGTGAAGTACGGGCAGAGCTGGGTGTTTGCAGCGGTGGCCTGCACAGGTGAGCTGCAGCTGGGCAGTCTGGGACCAATCTCAGCATGCAGTAGGCCC was designed to introduce the desired C-to-S mutation. The Johns Hopkins University Transgenic Core performed pronuclear injections of one-cell C57BL/6J embryos (Jackson Laboratories, Bar Harbor, ME, USA), using standard microinjection techniques [59] and a mix of Cas9 mRNA (10 ng/µL, ThermoFisher, Waltham, MA), sgRNA (5 ng/µL; IDT, Coralville, IA), and ssDNA HDR oligonucleotide (10 ng/µL; IDT) diluted in RNAse-free injection buffer (10 mM Tris-HCl, pH 7.4 and 0.25 mM EDTA). Injected embryos were transferred into pseudopregnant ICR females (Envigo, Indianapolis, IN) with the technique described by Nagy et al. [59].

### Genotyping protocol

Mouse genomic DNA was extracted from tail biopsies by using a tissue PCR Kit (REDExtract-N-Amp kit; Sigma-Aldrich), according to the manufacturer’s protocol. Two female founders were identified by Sanger sequencing of a PCR product flanking the targeted mutation site generated with the following primer pair: *Tgm2*—right: 5′-GAC TTT GAT CCC TTG CCG TA-3′ and *Tgm2*—left: 5′-GTC CAC CCA GAG ACT GGA AA-3′. For subsequent generations, an ARMS PCR assay was performed as follows: the *Tgm2* WT allele was identified using *Tgm2*—right 5′-GAC TTT GAT CCC TTG CCG TA-3′ and *Tgm2* WT—left 5′-GCT GCA AAC ACC CAG CA-3′. The mutant genotype was identified using *Tgm2*—right: 5′-GAC TTT GAT CCC TTG CCG TA-3′ and *Tgm2*-C277S mutant—left: 5′-GCT GCA AAC ACC CAG CT-3′. PCR conditions were as follows: initial denaturation at 94 °C for 30 s, 40 cycles at 94 °C for 30 s, 56 °C for 30 s, and 72 °C for 40 s, followed by final extension at 72 °C for 30 s. Both mutant and WT PCR protocols were performed for each tail biopsy. The presence of WT amplicon alone indicated a WT mouse, the presence of the mutant band alone indicated a homozygous mutant, and the presence of both indicated a heterozygous mouse.

### Aging study

*Tgm2*-C277S homozygous mutants and littermate WT mice were used. Age-matched TG2^−/−^ and corresponding Bl6/129S WT mice were used as controls. Genotyped mice were randomly assigned to young (3–6-month old) or old (>15-month old) cohort and used when they reached the appropriate age range. Each cohort comprised ten mice (ten females and ten males). The cohort sizes were based on power analysis to detect a 20% change in PWV with significance set at 0.05. PWV, an index of in vivo vascular stiffness, was measured noninvasively by using high-frequency Doppler (Indus Instruments), as previously described [48]. Investigators conducting data analysis and statistical evaluation were blinded to genotype and age of the groups. The aorta was then harvested and cut into 2-mm rings for tensile testing, wire myography, and western blotting. Additional mice were used for the ex vivo vascular and biochemical assays as indicated.

### Isolation and culture of VSMCs from mouse aorta

VSMCs were isolated as previously described [47, 48, 60]. Briefly, aorta was dissected out and cleaned free of connective tissue in ice-cold Krebs buffer supplemented with antibiotic-antimycotic. The cleaned aorta was cut into 2-mm rings and immediately placed in sterile complete media (DMEM, containing 10% FBS and antibiotic). A transverse cut was made and the endothelial layer was mechanically scraped off. The samples were immediately transferred to a cocktail of collagenase 2 (2 mg/mL, Worthington) in serum-free media and incubated at 37 °C for 2 h. Cells were collected by centrifugation, transferred to gelatin-coated dishes (35 mm), and maintained in 10% FBS. The identity of VSMCs was verified by the absence of PECAM-1 expression and the presence of smooth muscle actin (Fig. S1). Cells were used within two passages, and were serum-starved and tested for mycoplasma contamination before use.

### Cell adhesion and proliferation

Cell adhesion and spreading and cell proliferation were examined by using ECIS, as previously described [3, 61]. Briefly, for cell adhesion and spreading, 80,000 cells per well were seeded in uncoated or fibronectin-coated ECIS arrays (8W10E+; Applied Biophysics, Waltham, MA). The capacitive portion of impedance was measured at 40 kHz AC current until a stable plateau was reached in all samples. For cell proliferation, 40,000 cells per well were seeded in uncoated or fibronectin-coated ECIS arrays (8W10E+). Resistance at 4000 Hz AC current was measured until a stable plateau was reached in all samples. Adhesion and proliferation were examined with and without fibronectin coating (Millipore Sigma; catalog number F1141) of the culture surface. No TG2 was detected in the fibronectin used to coat cell culture ware (Fig. S2).

### DNA synthesis-based cell proliferation assay

We used the Click-iT EdU assay (ThermoFisher) according to the manufacturer’s protocol to identify DNA synthesis in proliferating cells [3]. Briefly, VSMCs were seeded on cell culture-treated coverslips at 30% confluence and serum-starved for 18 h. Next, cells were incubated for 24 h in complete growth medium containing EdU reagent (the modified thymidine analog 5-ethynyl-2′-deoxyuridine). Incorporation of EdU was determined by labeling with AlexaFluor azide 647, and total nuclei were labeled with DAPI. Coverslips were mounted and imaged by epifluorescence microscopy (Nikon 80i with CoolSnap HQ2). Four 10× images were obtained per coverslip and the fraction of EdU-positive to total nuclei was determined by using the object count function (Nikon NIS Elements Basic Science). Investigators that captured the images and performed data analysis were blinded to the VSMC genotype.

### GTP binding

Liver samples (100 mg wet tissue weight) from WT, TG2^−/−^, and *Tgm2*-C277S homozygous mice were homogenized in lysis buffer [1× radio-immuno-precipitation assay (RIPA) buffer containing protease inhibitor cocktail (Roche; Switzerland)]. Equal amounts of protein (1 mg) were withdrawn from each sample and brought to a volume of 500 µL with phosphate-buffered saline (PBS). Samples were incubated with GTP-agarose beads overnight at 4 °C with gentle rocking. After the beads were washed with PBS containing 300 mM NaCl, the bound proteins were eluted in 100 µL of a 10 mM GTP solution. The amount of TG2 bound to the beads was determined by western blotting. Cdc42 was used as a positive control for GTP binding. Polyclonal TG2 antibody was from Thermofisher and Cdc42 was from Abcam. Densitometry analysis was performed using ImageLab software (BioRad).

### TG2 activity assay

TG2 activity in the aorta was evaluated by three complementary methods described in prior publications [2–4, 47, 62]. First, we incubated intact aortic rings in biotin pentylamine (BPA), rinsed away unreacted PBA with PBS, and homogenized the tissue. We then used dot blotting to measure BPA incorporation into proteins. Second, we used aortic homogenates to examine the TG2-mediated crosslinking of FITC-cadaverine into *N*,*N*′-dimethylcasein by measuring increases in fluorescence polarization. And lastly, we determined FITC-cadaverine incorporation into the vascular media of intact aortic rings by en face confocal microscopy [22]. DTT (100 μM), a cell permeable reducing agent and a known activator of TG2’s transamidation function, was included in the ex vivo assays to identify the maximal TG2 activity in the specimens.

### Western blotting

Protein samples were prepared by homogenizing snap frozen tissue in 1× RIPA (ThermoFisher) buffer containing protease inhibitors (Roche). Protein concentrations were determined using the Bradford assay (BioRad protein assay reagent). Aliquots of total protein (10 μg for aorta and liver; 50 μg for all other tissues) resolved by SDS–PAGE and electro-transferred to nitrocellulose membranes. Blots were blocked with 5% nonfat dry milk in TBST for 1 h at room temperature and then incubated overnight with primary antibody. After rinsing, the membranes were incubated with HRP-conjugated secondary antibodies (BioRad) for 1 h at room temperature followed by chemiluminescent detection (BioRad ChemiDoc). Antibodies used were TG2 polyclonal (PA5-95256; ThermoFisher) and GAPDH (1D4; NB300-221; Novus Biologicals). ImageLab (BioRad) was used for densitometry analysis.

### Co-immunoprecipitation

TG2’s interaction with fibronectin and integrin β1 was determined by co-immunoprecipitation. Briefly, precleared liver homogenates (500 µg) were incubated with indicated antibody (0.5 µg) for 2 h at 4 °C with gentle rocking. The immunocomplexes were then enriched by incubating the samples with protein G-agarose beads (50 µL). The beads were rinsed three times to remove unbound proteins. Bound proteins were recovered by boiling the beads in 50 µL of 2× Laemmli buffer. Isotype antibody was used as a negative control. Immunoprecipitated proteins were resolved by SDS–PAGE, and immunocomplex-bound proteins were determined by western blotting. Antibodies used were fibronectin monoclonal (FBN11; Invitrogen), integrin β1 (CD29; HMβ1-1; BD Bioscience); and TG2 polyclonal (PA5-95256; ThermoFisher). Densitometry analysis was done using ImageLab software (BioRad).

### Pressure myography

Carotid artery compliance was measured by pressure myography, as previously described. Briefly, the carotid arteries of mice were dissected out, cleaned, and mounted in the chamber of a pressure myograph (DMT; Denmark) filled with oxygenated calcium-free Krebs buffer at 37 °C. After buffer was briefly passed through the vessel to evacuate blood remaining in the lumen, the vessel was equilibrated at 35 mmHg for 5 min followed by 50 mmHg for 15 min. Next, the pressure was increased by 10 mmHg every 2 min from 50 to 120 mmHg. Outer and inner diameter and wall thicknesses were recorded for each pressure level. Compliance pre- and post-remodeling was determined with the following equation:

$${\mathrm{Compliance}} = \frac{{{\Delta}V}}{{{\Delta}P}} \propto \frac{{{\Delta}Di^2}}{{{\Delta}P}}$$

where *V* is volume, *P* is pressure, and *Di* is lumen (inner) diameter.

### Wire myography

Vasoreactivity of isolated aortic rings was studied, as previously described [3, 4, 62]. Contraction response was determined with increasing concentrations of phenylephrine (10^−9^–10^−5^ mol/L) and normalized to a potassium chloride response (60 mM). Next, vessels were preconstricted with phenylephrine (10^−6^ mol/L; Sigma-Aldrich, St. Louis, MO) and endothelium-dependent and -independent vasorelaxation responses were determined with acetylcholine (10^−9^–10-5 mol/L) and sodium nitroprusside (10^−9^–10^−5^ mol/L; Sigma-Aldrich), respectively.

### Tensile testing

The elastic properties of intact and decellularized aortic rings were analyzed by tensile testing, as previously described [3, 4, 62]. Briefly, the thoracic aortae were harvested and cut into 2‐mm rings. Two intact and two decellularized rings from each animal were tested. We obtained transverse and longitudinal images of the rings to calculate vessel dimensions [lumen diameter (*Di*), wall thickness (*t*), and length (*L*)], and then mounted the rings on an electromechanical puller (DMT). After calibration and alignment, we moved the pins apart using an electromotor and recorded displacement and force continuously. Engineering stress (*S*) was calculated by normalizing force (*F*) to the initial stress‐free area of the specimen (*S* = *F*/2*t* × *L*; where *t* = thickness and *L* = length of the sample). Engineering strain (*λ*) was calculated as the ratio of displacement to the initial stress‐free diameter. The stress–strain relationship was represented by the equation *S* = α exp (*βλ*), where *α* and *β* are constants. *α* and *β* were determined by nonlinear regression for each sample and used to generate stress–strain curves by treating the *x*‐axis as a continuous variable. Incremental elastic modulus (*E*_inc_) was calculated as the slope of the stress–strain curve at a strain of 0.5 and 1.8.

### Statistical analysis

Data are presented as arithmetic mean ± standard error of the mean. Sample size (*n*) is indicated for each reported value. For statistical evaluation, two means were compared by the Student’s *t* test and groups were compared with one-way analysis of variance (ANOVA) with Bonferroni’s post hoc analysis. For multiple comparisons, two-way ANOVA with Bonferroni post hoc analysis was used. Means were considered to be statistically different at *p* < 0.05.

## Supplementary information

Supplemental Figure 1

Supplemental Figure 2

Author contribution form
