# Supplementary figures and images for: Probing tissue transglutaminase mediated vascular smooth muscle cell aging using a novel transamidation-deficient Tgm2-C277S mouse model
Source: Cell Death Discov. 2021 Jul 29;7:197. doi: 10.1038/s41420-021-00543-8 (PMC8322091; doi:10.1038/s41420-021-00543-8)

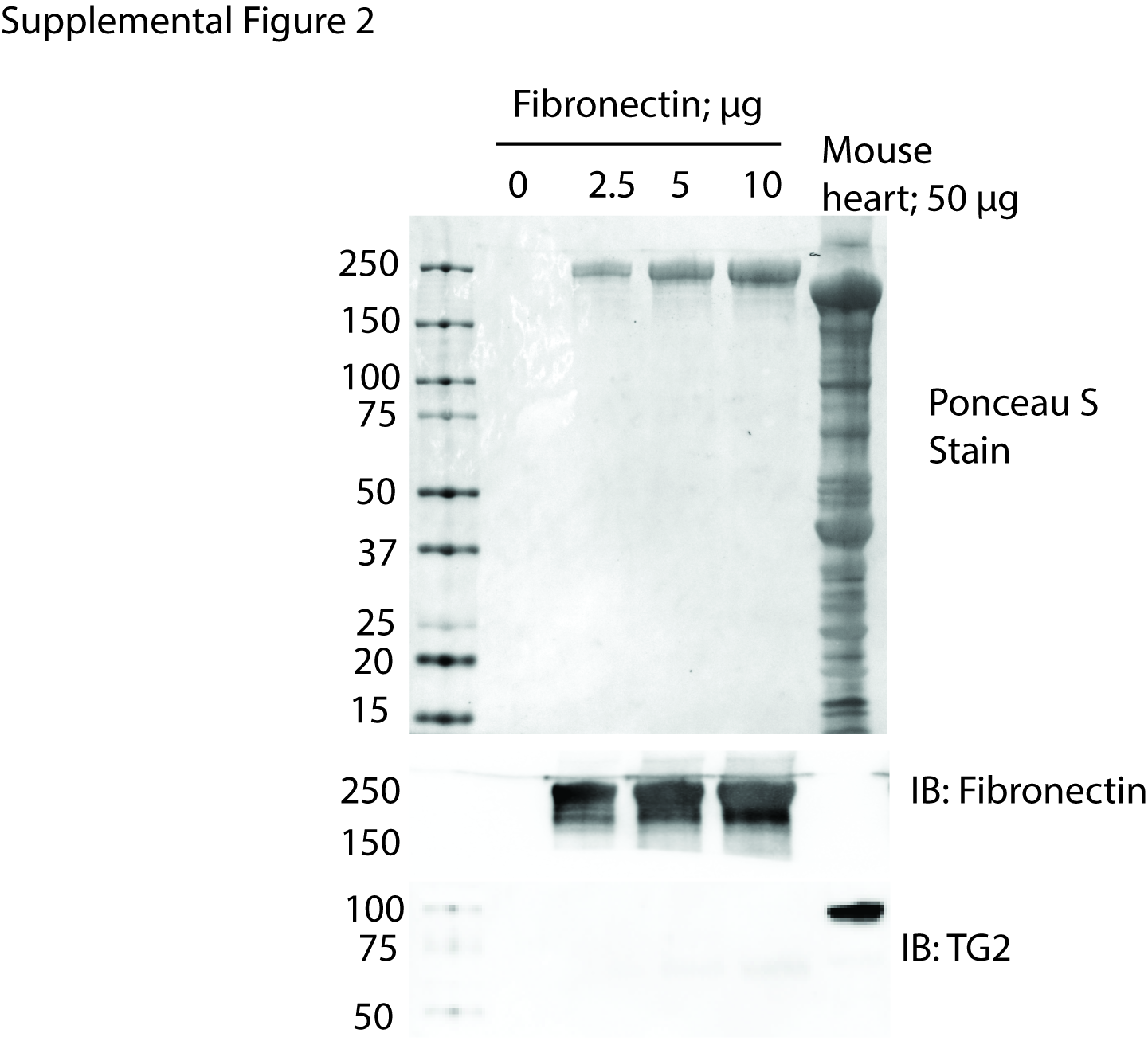

Supplement: Supplementary file 2 — Supplemental Figure 2 [file 41420_2021_543_MOESM2_ESM.tif]
