# Supplementary material for: Probing tissue transglutaminase mediated vascular smooth muscle cell aging using a novel transamidation-deficient Tgm2-C277S mouse model
Source: Cell Death Discov. 2021 Jul 29;7:197. doi: 10.1038/s41420-021-00543-8 (PMC8322091; doi:10.1038/s41420-021-00543-8)
Supplement: Supplementary file 3 — Author contribution form [file 41420_2021_543_MOESM3_ESM.pdf]

DECLARATION OF CONTRIBUTIONS TO ARTICLE

ADMC

Manuscript Number:	Journal Name:	
CDDISCOVERY-20-1298-T	*Cell Death Discovery*	(the 'Journal')

Proposed Title of the Contribution:

Probing tissue transglutaminase mediated vascular smooth muscle cell aging using a novel transamidation-deficient TGM2-C277S mouse model (the 'Contribution)

Author(s):

Huilei Wang, James Chen; Sandeep Jandu, Sean Melucci, William Savage, Kavitha Nandakumar, Sara K. Kang, Sebastian Barreto-Ortiz, Alan Poe, Shivam Rastogi, Maria Bauer, Jochen Steppan, and Lakshmi Santhanam (the 'Authors')

For all *CDDis*covery articles, each person named as an author in the published version must be able to show he or she has contributed substantially to the article.

Authorship credit should be based on 1) substantial contributions to conception and design, acquisition of data, or analysis and interpretation of data; 2) drafting the article or revising it critically for important intellectual content; and 3) final approval of the version to be published. Authors should meet conditions 1, 2 and 3.

Any person who cannot be shown to have made a substantial contribution to the article cannot be listed as an author in the final version. The name of any person who is deemed to have made a minor contribution can, however, appear in the Acknowledgments section of the article.

Please complete the table below to indicate the contributions of all named authors to the manuscript.

Author Full Name:	Specification of Contribution to the Manuscript:
Huilei Wang	Acquisition, analysis, interpretation of data
James Chen	Acquisition, analysis, interpretation of data
Sandeep Jandu	Acquisition, analysis, interpretation of data
Sean Melucci	Acquisition, analysis, interpretation of data
William Savage	Acquisition, analysis of data
Kavitha Nandakumar	Acquisition, analysis of data
Sara K Kang	Data acquisition
Sebastian Barreto-Ortiz	Data acquisition
Alan Poe	Data acquisition
Shivam Rastogi	Data acquisition
Maria Bauer	Data acquisition
Jochen Steppan	Data acquisition, analysis, funding
Lakshmi Santhanam	Design of study, provided funding, drafted and revised manuscript, data analysis and interpretation, final approval

Please complete the table below to indicate the contributions of all named authors to the figures.

Figure 1:

Sandeep Jandu, Kavitha Nandakumar, Lakshmi Santhanam

Figure S1: Huilei Wang, Alan Poe

Figure 2:

Huilei Wang, Sean Melucci, James Chen

Figure S2: Huilei Wang, Alan Poe

Figure 3:

Huilei Wang, James Chen, Sebastian Barreto-Ortiz

Figure 4:

Sean Melucci, Huilei Wang, James Chen, Sandeep Jandu, Sara Kang

Figure 5:

Huilei Wang, James Chen, Sandeep Jandu

Figure 6:

Sean Melucci, James Chen, Sandeep Jandu, Shivam Rastogi, Sara Kang, William Savage, Maria Bauer, Jochen Steppan

Figure 7: James Chen, Huilei Wang, Alan Poe, Kavitha Nandakumar

Signed for and on behalf of the Author(s):	Print Name:	Date:
Lakshmi Santhanam	Lakshmi Santhanam	4/6/2021
